# Effect of *Allium cepa* L. on Lipopolysaccharide-Stimulated Osteoclast Precursor Cell Viability, Count, and Morphology Using 4′,6-Diamidino-2-phenylindole-Staining

**DOI:** 10.1155/2014/535789

**Published:** 2014-08-03

**Authors:** Tatiane Oliveira, Camila A. Figueiredo, Carlos Brito, Alexander Stavroullakis, Anuradha Prakki, Eudes Da Silva Velozo, Getulio Nogueira-Filho

**Affiliations:** ^1^Department of Bioregulation, Institute of Health Sciences, Federal University of Bahia, 41110-100 Salvador, BA, Brazil; ^2^Department of Biological and Diagnostic Sciences-Preventive Dentistry, University of Toronto, Toronto, ON, Canada M5G 1G6; ^3^Department of Clinical Sciences-Restorative, University of Toronto, Toronto, ON, Canada M5G 1G6; ^4^Department of Medicines, Faculty of Pharmacy, Federal University of Bahia, 40170-115 Salvador, BA, Brazil

## Abstract

*Allium cepa* L. is known to possess numerous pharmacological properties. Our aim was to examine the *in vitro* effects of *Allium cepa* L. extract (AcE) on *Porphyromonas gingivalis* LPS and *Escherichia coli* LPS-stimulated osteoclast precursor cells to determine cell viability to other future cell-based assays. Osteoclast precursor cells (RAW 264.7) were stimulated by *Pg* LPS (1 *μ*g/mL) and *E. coli* LPS (1 *μ*g/mL) in the presence or absence of different concentrations of AcE (10–1000 *μ*g/mL) for 5 days at 37°C/5% CO_2_. Resazurin reduction and total protein content assays were used to detect cell viability. AcE did not affect cell viability. Resazurin reduction assay showed that AcE, at up to 1000 *μ*g/mL, did not significantly affect cell viability and cellular protein levels. Additionally a caspase 3/7 luminescence assay was used to disclose apoptosis and there was no difference in apoptotic activity between tested groups and control group. Fluorescence images stained by DAPI showed no alteration on the morphology and cell counts of LPS-stimulated osteoclast precursor cells with the use of AcE in all tested concentrations when compared to control. These findings suggest that *Allium cepa* L. extract could be used for *in vitro* studies on *Porphyromonas gingivalis* LPS and *Escherichia coli* LPS-stimulated osteoclast precursor cells.

## 1. Introduction

The natural products derived from medicinal plants have proven to be an abundant source of biologically active compounds and some of these extracts are considered to be beneficial to health [1]. Scientific evaluation of plant's properties through potent pharmacological activities, toxicity profiling, and economic viability is needed for enhancing recognition of medicinal plants and herbal products [2].

Recently, studies with natural compounds have advanced significantly showing the modulation of the immune system in pathological processes [3–5]. Inflammation is an important host response to foreign challenge or tissue injury, which leads to the restoration of tissue structure and function [6]. However, prolonged inflammation can cease to be a beneficial event, and it contributes to the pathogenesis of many disease states, such as asthma, diabetes, rheumatoid arthritis, and periodontitis [7, 8].

These diseases have different initiation factors; however, all are mediated immune disorders that induce tissue lesion, which can result in complex biological effects* in vivo* [6–8]. Lipopolysaccharide (LPS), a component of the cell walls of gram-negative bacteria, is a microbial factor contributing to inflammatory diseases [9–11].* Allium cepa* L. has been studied and associated with antiallergic potential in bronchial pathology [5] as well as antibacterial, antihistaminic, anti-inflammatory, and antioxidant activities [12]. However, the increasing interest in natural compounds has led to increased attention to their safety profiles and toxicity [9]. A limited number of these compounds have sufficiently been studied and their efficacy remains to be investigated [3]. Thus, the aim of the present study was to determine the effect of a methanolic extract of* Allium cepa* L. on cell viability, cell morphology, induction of apoptosis, cell count of osteoclast precursor cells (RAW 264.7), and proliferation under* Porphyromonas gingivalis* (*Pg*) LPS and* Escherichia coli* LPS-stimulated inflammatory conditions. The idea is to determine the non-cytotoxic concentrations and viable cell number for future other cell-based assays.

## 2. Material and Methods

Resazurin reduction, protein assays, caspase 3/7 luminescence assay, and fluorescence images stained by DAPI were the methods used to determine the noncytotoxic concentrations of* Allium cepa* L. on the RAW 264.7 osteoclast precursor cell line.

### 2.1. Reagents

*Allium cepa* L. samples were peeled cut and extracted by maceration in flasks containing 1000 mL of 99.8% methyl alcohol (CH_3_OH) for 7 days. After filtering, the extract was concentrated by evaporation under reduced pressure using a rotary evaporator. This concentration process was repeated three times. Subsequently, the AcE was dried in the oven and then maintained at −20°C until use. Different concentrations of AcE (10, 50, 100, or 1000 *μ*g/mL) were tested. LPS from* Escherichia coli*,* Porphyromonas gingivalis*, dimethyl sulfoxide (DMSO) ≥99.5%, and the other chemicals were purchased from Sigma Aldrich (St. Louis, MO, USA).

### 2.2. Cell Culture

Murine monocyte/macrophage RAW 264.7 cells (ATCC accession no. TIB-71) were cultured and maintained at 37°C in a 5% CO_2_ humidified atmosphere with DMEM (Invitrogen 11995) that was supplemented with 10% FBS (Invitrogen 12484028) and 100 *μ*g/mL penicillin/streptomycin. The medium was changed every 3 days.

### 2.3. Cell Toxicity Assays

Cells were grown in 96-well plates with* Pg* LPS and* E. coli* LPS-stimulation in the presence and absence of AcE (10–1000 *μ*g/mL). DMSO (50%) was used for positive control. Resazurin reduction assays (Alamar blue) (Invitrogen, DAL 1025) were performed after 5 days. The cultures were placed in medium containing 10% of Alamar blue. After 4 h of incubation, 100 *μ*L of the medium was transferred to the wells of a 96-well plate and the optical density (OD) was measured using Cytation 3, Imaging reader (Biotek, USA) at wavelengths of 570 and 600 nm. The percentage of cells showing cytotoxicity relative to the control group (Ctrl) was determined. A greater percentage of reduction on Alamar blue assay reflects greater cell proliferation. Additionally, protein assays were used to evaluate cell proliferation. Cell lysis was carried out as listed in Kartner et al., 2010 [13]. Cells were briefly washed with PBS and lysed with protein lysis buffer (90 mM trisodium citrate, 10 mM NaCl, 0.1% Triton X-100, pH 4.8). Protein concentrations were determined using the Pierce BCA protein assay (Thermo Scientific 23225). The OD was measured using Cytation 3, Imaging reader (Biotek, USA) at 562 nm wavelengths that provided the formula to calculated the protein levels.

### 2.4. Caspase 3/7 Assay

Activities of caspase-3/7 were measured by a Caspase-Glo 3/7 assay kit (Pierce, USA). Cells were cultured in the presence and absence of AcE (10–1000 *μ*g/mL) for 5 days and were added with 100 *μ*L of Caspase-Glo 3/7 Reagent. After mixing gently and incubating the cells at room temperature for 1 h, the luminescence of each sample was measured in the plate-reading luminometer of Cytation 3, Biotek (USA).

### 2.5. Image Analysis and Cell Count by DAPI Staining

Cells were cultured with* Pg* LPS and* E. coli* LPS-stimulation in the presence and absence of AcE (10–1000 *μ*g/mL) for 5 days. DMSO (50%) was used for positive control. Cells were stained with 4,6-diamidino-2-phenylindole (DAPI, Sigma-Aldrich) and then automatically counted. Five visual images were taken using fluorescent microscopy (Cytation 3, Biotek, USA). Fragmented or condensed nuclei were defined as apoptotic cells [14] and each sample was analyzed using Gen5 software (Biotek, USA).

### 2.6. Statistical Analysis

Statistical significance (*P* ≤ 0.05) was determined by ANOVA; the data were further analyzed by Tukey's pair wise comparisons to detect specific differences between tested groups and control group using GraphPad Prism (GraphPad Software Inc., San Diego, CA, USA). Each experiment was carried out in triplicate and repeated at least three times.

## 3. Results and Discussion

This study investigated the effect of extracts of* Allium cepa* L. (AcE) on cell viability in LPS-stimulated osteoclast precursor cells. Our results showed that treatment with various concentrations of AcE had no significant toxic effects meaning that cells were viable under the experimental conditions evaluated.

Inflammation triggered by infection and tissue injury underlies a variety of physiological and pathological processes [6]. There has been growing interest to investigate pharmacological activities from natural products in mediated immune pathologies such as periodontitis [3, 9]. However, a limited number of natural compounds have sufficiently been studied in relation to efficacy and safety [9, 15].

The production of inflammatory molecules by murine monocyte macrophage RAW 264.7 cells can be induced in response to LPS stimulation [9, 11]. Thus, inhibitors of these inflammatory molecules may be considered anti-inflammatory drug candidates [16].* Allium cepa* L. has been used to treat inflammatory conditions such as asthma [5] and ovariectomy-induced bone resorption in rats [17]. AcE inhibits RANKL-induced ERK, p38, and NF-*κ*B activation in osteoclast precursor cells from rats [18].

**Figure 1:**
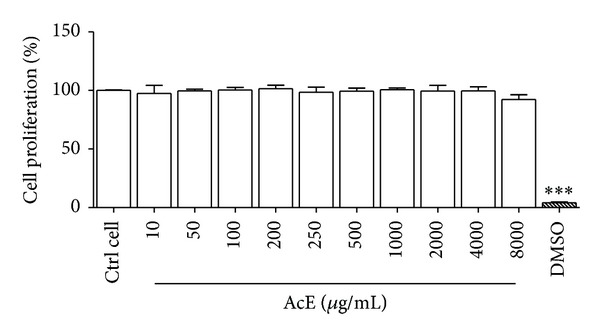
Effect of* Allium cepa* L. on cell proliferation on RAW 264.7 cells. Cells were cultured in the presence of AcE (10–8000 *μ*g/mL); untreated cells (Ctrl) were not exposed to AcE; and DMSO was used for positive control. After 5 days of continuous exposure, cell proliferation was assessed using Alamar blue assay. The columns represent the mean values of the results obtained of three independent experiments. There was no significance between negative control (Ctrl) and cells in all concentrations tested. There was significance between positive control (DMSO) and cells in all concentrations tested *P* < 0.001*** ANOVA-Tukey.

**Figure 2:**
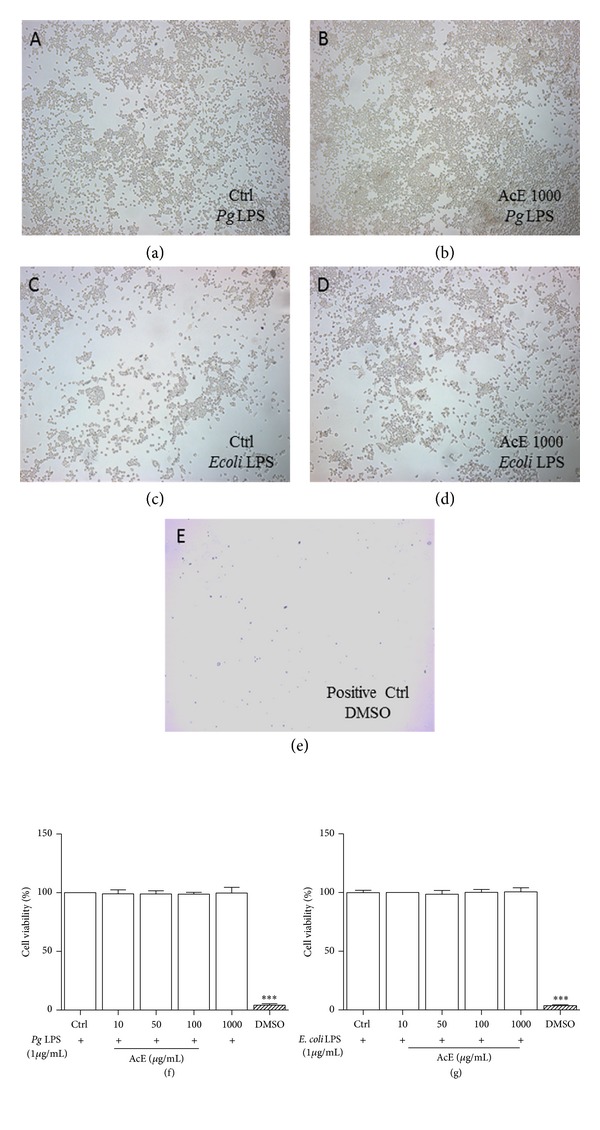
Effect of* Allium cepa* L. on cell viability on* Pg* LPS and* E. coli* LPS-induced RAW 264.7 cells. Cells were cultured in the presence of* Pg* LPS ((a), (b), and (f)) or* E. coli* LPS ((c), (d), and (g)), and AcE; untreated cells (Ctrl) were not exposed to AcE and DMSO was used for positive control (e). After 5 days of continuous exposure, cell proliferation was assessed using Alamar blue assay. Photographs shown are representative of results observed in multiple photographs taken for the following groups: Ctrl* Pg* LPS (a) and AcE 1000 *μ*g/mL (b); Ctrl* E. coli* LPS (c) and AcE 1000 *μ*g/mL (d). In (f) and (g) the columns represent the mean values of the results obtained of three independent experiments. There was no significance between control (Ctrl) and cells in all concentrations tested. There was significance between positive control (DMSO) and cells in all concentrations tested *P* < 0.001*** ANOVA-Tukey.

**Figure 3:**
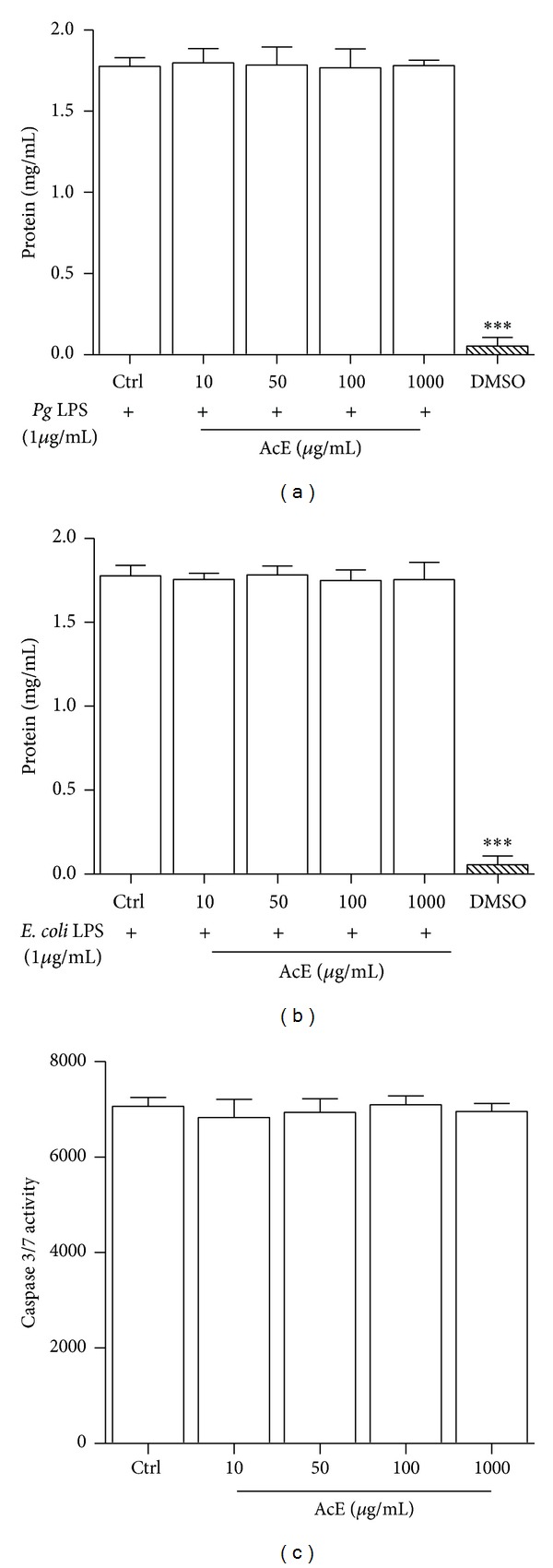
Effect of* Allium cepa* L. on total protein levels of LPS-induced and apoptosis in RAW 264.7 cells. In (a) and (b), cells were cultured in the presence of* Pg* LPS (a) and* E. coli* LPS (b), and AcE; untreated cells (Ctrl) were not exposed to AcE. DMSO was used for positive control. After 5 days of continuous exposure, quantification of total protein levels was assessed using protein assays. In (c), activities of caspase-3 and caspase-7 were examined by caspase 3/7 assay. In (a), (b), and (c) columns represent the mean values of the results obtained of three independent experiments, and error bars represent the standard error from the means. There was no significance between control (Ctrl) and cells in all concentrations tested, ANOVA-Tukey.

Cell-based assays are used to determine if the tested drugs have pharmacological effects on cell proliferation or show direct cytotoxicity effects that eventually lead to cell death [19]. Various techniques have been used to investigate cell viability. In this study the resazurin-reduction, protein assays, and morphology and cell count by DAPI staining were used. Dimethyl sulfoxide (DMSO) is a cell culture reagent with many applications in chemical and biological research [19]. In this study it was used for comparison (positive control). Additionally, cleaved caspase-3 staining was used to identify apoptosis in RAW 264.7 cell. Resazurin is a cell permeable redox indicator that can be used to monitor viable cell number with protocols similar to those utilizing the tetrazolium compounds. However, it is slightly more sensitive than tetrazolium reduction assays (MTT). Viable cells with active metabolism can reduce resazurin into the resorufin product that is pink and fluorescent [20]. In this study, according to the Alamar blue assay, the exposure of RAW 264.7 cells to different concentrations of* Allium cepa* L. over a period of 5 days was not significantly affected, suggesting that AcE did not have a proliferative effect, and IC_50_ value is higher than 8000 *μ*g/mL (Figure 1). In Addition, the exposure of* Pg* LPS or* E. coli* LPS-stimulated RAW 264.7 cells to AcE at up to 1000 *μ*g/mL is not toxic to this specific cell line (Figures 2(a)–2(g)). Additionally, to confirm the effect of AcE on cell viability the protein concentration in all samples were compared to a protein standard. This test combines the well-known reduction of Cu^2+^ to Cu^1+^ by protein in an alkaline medium with the highly sensitive and selective colorimetric detection of the cuprous cation (Cu^1+^) by bicinchoninic acid [21]. In order to quantify total protein levels protein assays were used. Over a period of 5 days under the same growth conditions the* Pg* LPS and* E. coli* LPS-stimulated cells did not show any significant reduction in total protein levels at up to 1000 *μ*g/mL when compared to negative control group (Ctrl) as shown in Figures 3(a) and 3(b). Apoptotic cell death in RAW 264.7 macrophages and several other systems may be the result of cytotoxicity induced by the cell treatment with different drugs and through the upregulation of activation of caspases [22, 23]. Apoptosis was not induced by the treatment of AcE, at up to 1000 *μ*g/mL (Figure 3(c)).

**Figure 4:**
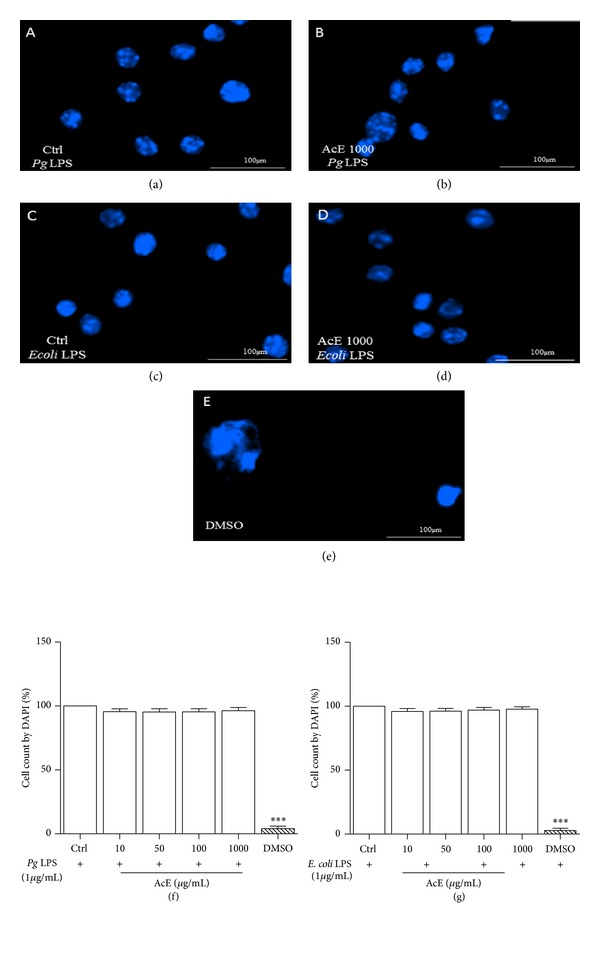
Effect of* Allium cepa* L. on cell morphology and cell count by DAPI on LPS-induced RAW 264.7 cells. RAW cells were cultured in the presence of* Pg* LPS ((a), (b), and (f)) or* E. coli* LPS ((c), (d), and (g)), and AcE; untreated cells (Ctrl) were not exposed to AcE. In (a) (Ctrl* Pg* LPS) and (b) (AcE 1000 *μ*g/mL); (c) (Ctrl* E. coli* LPS) and (d) (AcE 1000 *μ*g/mL) morphology was observed. DMSO was used for positive control (e). In (f) and (g), cell count by DAPI staining using a fluorescence microscope. Columns represent the mean values of the results obtained of three independent experiments. There was no significance between control (Ctrl) and cells in all concentrations tested, ANOVA-Tukey.

DAPI is a DNA-specific probe which forms a fluorescent complex by attaching in the minor grove of A-T rich sequences of DNA and was first synthesized in Otto Dann's laboratory at Erlangen [24]. The practicality of DAPI for fluorescent microscopic observation of DNA in bacteria, plant, protozoa, and mammalian cells was demonstrated in that study. These observations established DAPI as a DNA-specific fluorescent stain [25]. Thus, under the same growth conditions, DAPI staining did not show any reduction in the number of cells or alteration in the morphology in all AcE tested concentrations when compared to negative control group (Ctrl) (Figures 4(a)–4(g)).

In conclusion, the findings of the present study provided adequate evidence that* Allium cepa* L. methanol extract can be safely used in further pharmacological investigations utilizing* Pg* and* E. coli* LPS-stimulated osteoclast precursor cells.
